# Supplementary material for: Telomeric 8-oxo-guanine drives rapid premature senescence in the absence of telomere shortening
Source: Nat Struct Mol Biol. 2022 Jun 30;29(7):639–52. doi: 10.1038/s41594-022-00790-y (PMC9287163; doi:10.1038/s41594-022-00790-y)
Supplement: Supplementary file 1 — Reporting Summary [file 41594_2022_790_MOESM1_ESM.pdf]

nature portfolio

Corresponding author(s): Patricia L Opresko

Last updated by author(s): Apr 4, 2022

Reporting Summary

Nature Portfolio wishes to improve the reproducibility of the work that we publish. This form provides structure for consistency and transparency in reporting. For further information on Nature Portfolio policies, see our Editorial Policies and the Editorial Policy Checklist.

Statistics

For all statistical analyses, confirm that the following items are present in the figure legend, table legend, main text, or Methods section.

n/a	Confirmed	
☐	☒	The exact sample size (*n*) for each experimental group/condition, given as a discrete number and unit of measurement
☐	☒	A statement on whether measurements were taken from distinct samples or whether the same sample was measured repeatedly
☐	☒	The statistical test(s) used AND whether they are one- or two-sided<br>*Only common tests should be described solely by name; describe more complex techniques in the Methods section.*
☒	☐	A description of all covariates tested
☒	☐	A description of any assumptions or corrections, such as tests of normality and adjustment for multiple comparisons
☐	☒	A full description of the statistical parameters including central tendency (e.g. means) or other basic estimates (e.g. regression coefficient) AND variation (e.g. standard deviation) or associated estimates of uncertainty (e.g. confidence intervals)
☐	☒	For null hypothesis testing, the test statistic (e.g. *F*, *t*, *r*) with confidence intervals, effect sizes, degrees of freedom and *P* value noted<br>*Give P values as exact values whenever suitable.*
☒	☐	For Bayesian analysis, information on the choice of priors and Markov chain Monte Carlo settings
☒	☐	For hierarchical and complex designs, identification of the appropriate level for tests and full reporting of outcomes
☒	☐	Estimates of effect sizes (e.g. Cohen's *d*, Pearson's *r*), indicating how they were calculated

Our web collection on statistics for biologists contains articles on many of the points above.

Software and code

Policy information about availability of computer code

Data collection

NIS Elements AR 5.1 Nikon
Prism 9 Graph Pad
Accuri C6 Software BD
ImageQuant TL GE 8.2

Data analysis

RNA sequencing data was aligned to the transcriptome with Salmon (V0.7.2) the resulting counts were analyzed with DEseq2 (V1.30.1) and enrichments calculated with FGSEA (V1.16.0), Matlab (2017A) was used for sorting genes by chromosomal coordinates and plotting

For manuscripts utilizing custom algorithms or software that are central to the research but not yet described in published literature, software must be made available to editors and reviewers. We strongly encourage code deposition in a community repository (e.g. GitHub). See the Nature Portfolio guidelines for submitting code & software for further information.

Data

Policy information about availability of data

All manuscripts must include a data availability statement. This statement should provide the following information, where applicable:

- Accession codes, unique identifiers, or web links for publicly available datasets
- A description of any restrictions on data availability
- For clinical datasets or third party data, please ensure that the statement adheres to our policy

All relevant data are available in the Source Data files or from the authors upon reasonable request. The mRNAseq dataset are deposited at GEO (GSE175686).

Field-specific reporting

Please select the one below that is the best fit for your research. If you are not sure, read the appropriate sections before making your selection.

☒ Life sciences ☐ Behavioural & social sciences ☐ Ecological, evolutionary & environmental sciences

For a reference copy of the document with all sections, see nature.com/documents/nr-reporting-summary-flat.pdf

Life sciences study design

All studies must disclose on these points even when the disclosure is negative.

Sample size	No sample-size calculations were performed. Sample sizes were chosen based on similar published studies and standards in the field and experience. The sample size (n) is given in the figure legends.
Data exclusions	No data were excluded from the analyses, except for rare outliers as identified by GraphPad Prism 9 software, and indicated as blue in the Source Data.
Replication	Independent experiments were conducted a minimum of 3 times unless otherwise noted in the figure legends. All replications were successful.
Randomization	No randomization was performed. Randomization and covariates are not applicable here since we used genetically defined cell lines cultured at the same time under the same conditions, to increase robustness and control for experimental variation.
Blinding	Blinding was not done. Automated image analysis and quantitation was used to minimize bias.

Reporting for specific materials, systems and methods

We require information from authors about some types of materials, experimental systems and methods used in many studies. Here, indicate whether each material, system or method listed is relevant to your study. If you are not sure if a list item applies to your research, read the appropriate section before selecting a response.

Materials & experimental systems

n/a	Involved in the study	
☐	☒	Antibodies
☐	☒	Eukaryotic cell lines
☒	☐	Palaeontology and archaeology
☒	☐	Animals and other organisms
☒	☐	Human research participants
☒	☐	Clinical data
☒	☐	Dual use research of concern

Methods

n/a	Involved in the study	
☒	☐	ChIP-seq
☐	☒	Flow cytometry
☒	☐	MRI-based neuroimaging

Antibodies

Antibodies used

GFP Abcam ab6556 (1:1000 WB and IF)
TRF1 Abcam ab10579 (1:200 WB)
GAPDH Santa Cruz sc-47724 (1:30000 WB)
OGG1 Abcam Ab124741 (1:500)
Actin Cell Signaling 3700S (1:30000 WB)
LaminB1 Abcam ab16048 (1:500 WB and IF)
LaminA/C Cell Signaling 4777S (1:500 WB and IF)
γH2AX Santa Cruz sc-517348 (1:1000 WB, 1:250 IF)
53BP1 Novus NB100-304 (1:1000 IF)
TRF2 Novus NB110-57130 (1:500 IF)
MDM2 Cell Signaling 86934S (1:1000 WB)
p53 Santa Cruz sc-126 (1:200 WB)
p21 Cell Signaling 2947S (1:1000 IF and 1:2000 WB)
p16 Proteintech 10883-1-AP (1:200 WB)
pRB S807/811 Cell Signaling 8516S (1:500 WB)
pCHK2 T68 Cell Signaling 2197S (1:1000 WB)
pCHK1 S317 Cell Signaling 12302S (1:500 WB)
pATM S 1981 Abcam ab81292 (1:2000 WB)
CHK1 Cell Signaling 2360S (1:1000 WB)

Chk2 Cell Signaling 3440 (1:1000 WB)
H3K27me3 Cell Signaling 9733 (1:500 IF)
H3K27Ac Cell Signaling 8173 (1:500 IF)
LSD1 Cell Signaling 2184 (1:500 IF)
cGAS Cell Signaling 66546 (1:200 IF)
p62 Cell Signaling 39749 (1:500 IF)

Secondary antibodies
Anti-Rabbit IgG HRP Secondary, Sigma A0545 (1:20000 WB)
Goat Anti-mouse IgG HRP antibody, Sigma A0168 (1:20000 WB)
Goat anti rabbit IgG (H+L) secondary ab Alexa fluor 488,Thermo PIA32731 (1:500 IF)
Goat anti-mouse IgG secondary Ab, Alexa Fluor 594, Thermo A32742 (1:500 IF)
Goat anti-mouse IgG secondary Ab, Alexa Fluor 647, Thermo A32728 (1:500 IF)
Goat anti-rabbit IgG secondary, Alexa Fluor 594, Thermo A32740 (1:500 IF)
Alexa Fluor 647 AffiniPure F(ab')2 Goat anti-rabbit antibody, Jackson Labs 111-606-045 (1:500 IF)

Validation

Antibodies were validated either by the manufacturer as indicated on the corresponding websites, and/or by this study in cell lines knocked out or knocked down for the gene product (OGG1, p16 and p53).

Eukaryotic cell lines

Policy information about cell lines

Cell line source(s)

hTERT BJ ATCC CRL-4001
hTERT RPE ATCC CRL-4000
Primary BJ ATCC CRL-2522
HeLa LT O'Sullivan lab
U2OS ATCC HTB-96
HEK29T ATCC CRL-3216

Authentication

Authentication done by ATCC for cell lines obtained from ATCC, including STR profiling. HeLa LT cell lines was not authenticated by us for this study.

Mycoplasma contamination

All cell lines tested negative for mycoplasma contamination as confirmed by DAPI staining and microscopy, and MycoAlert elisa assay.

Commonly misidentified lines
(See ICLAC register)

None

Flow Cytometry

Plots

Confirm that:

☒ The axis labels state the marker and fluorochrome used (e.g. CD4-FITC).

☒ The axis scales are clearly visible. Include numbers along axes only for bottom left plot of group (a 'group' is an analysis of identical markers).

☐ All plots are contour plots with outliers or pseudocolor plots.

☒ A numerical value for number of cells or percentage (with statistics) is provided.

Methodology

Sample preparation

Cells were detached with trypsin and processed live for apoptosis measure, or fixed for cell cycle analysis.

Instrument

Accuri C6 (Beckman)

Software

Accuri C6 (Beckman)

Cell population abundance

All cells analyzed

Gating strategy

Cells gated from debris with FSC/SSC. Singlets gated with FSC-A/FSC-H.

☒ Tick this box to confirm that a figure exemplifying the gating strategy is provided in the Supplementary Information.
