# Supplementary figures and images for: Telomeric 8-oxo-guanine drives rapid premature senescence in the absence of telomere shortening
Source: Nat Struct Mol Biol. 2022 Jun 30;29(7):639–52. doi: 10.1038/s41594-022-00790-y (PMC9287163; doi:10.1038/s41594-022-00790-y)

Figure 1b

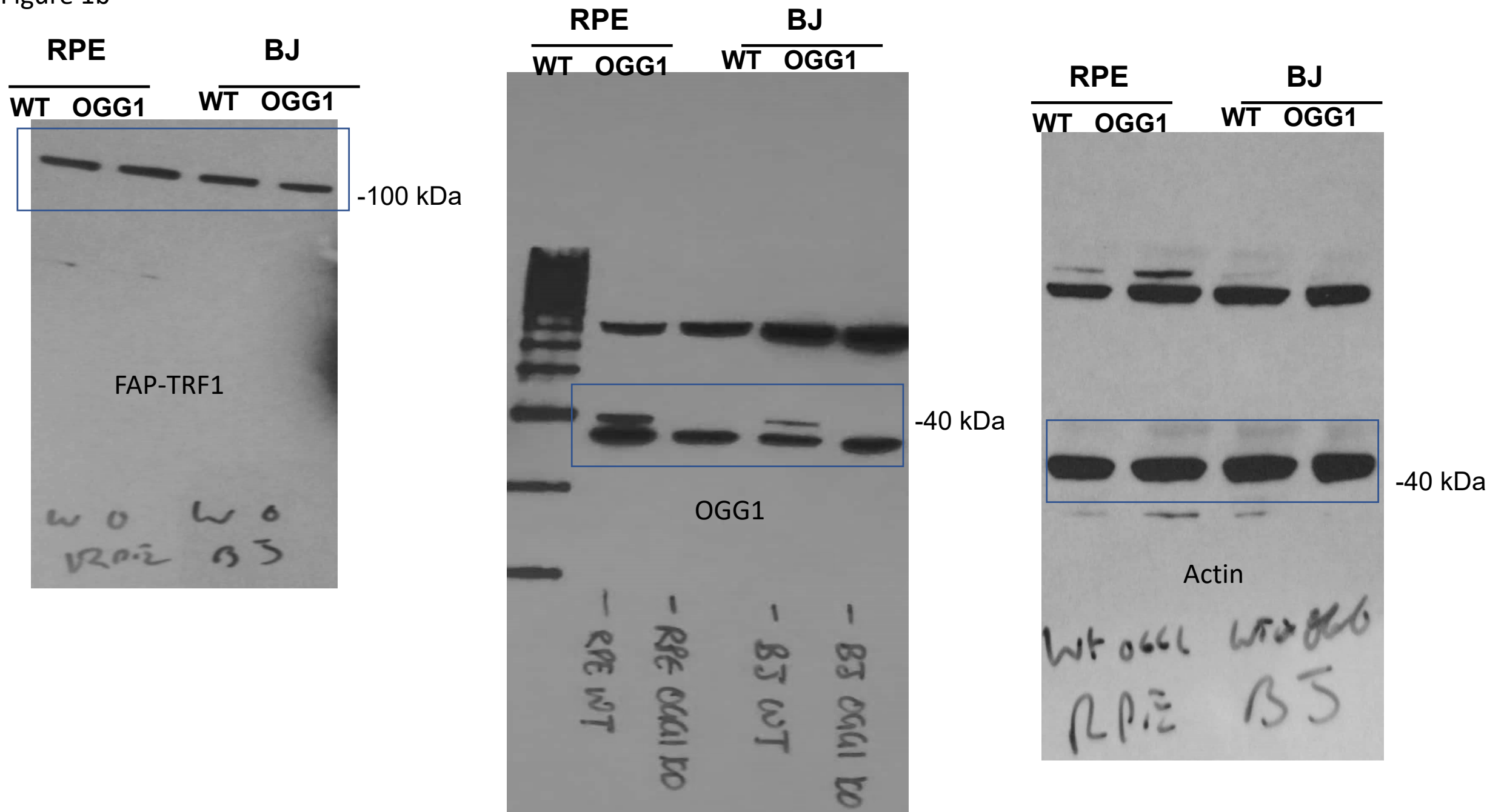

Supplement: Source Data Fig. 1 — Unprocessed western blots. [file 41594_2022_790_MOESM8_ESM.pdf]

Fig 3a

RPE

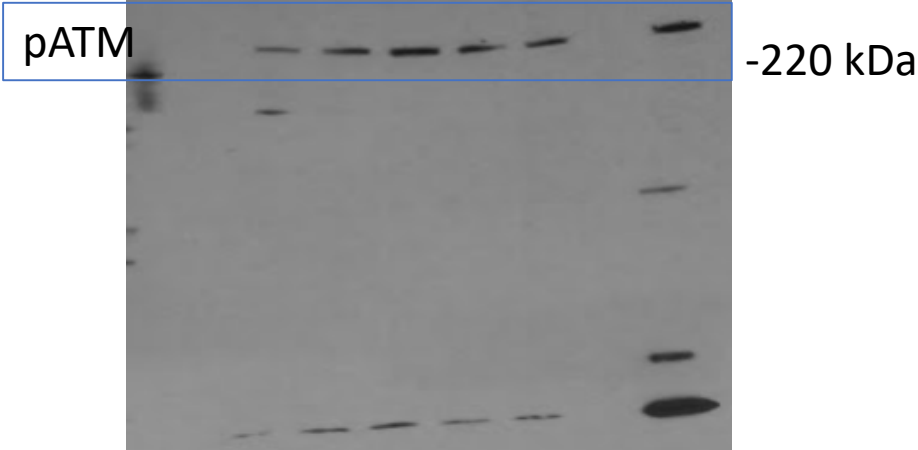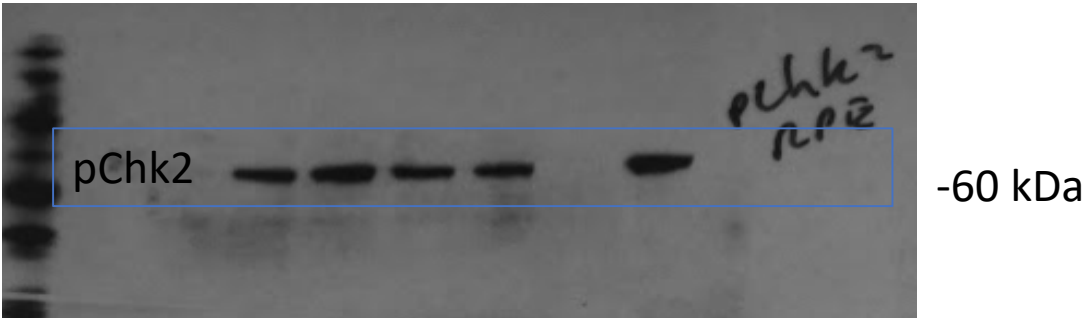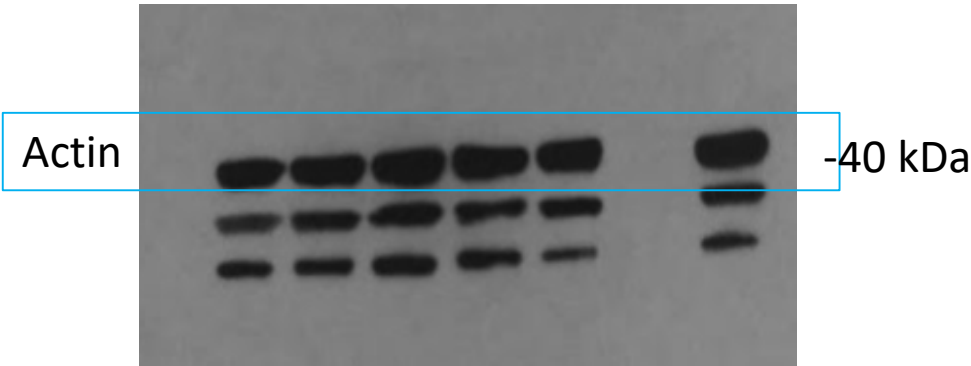

Fig 3a

BJ

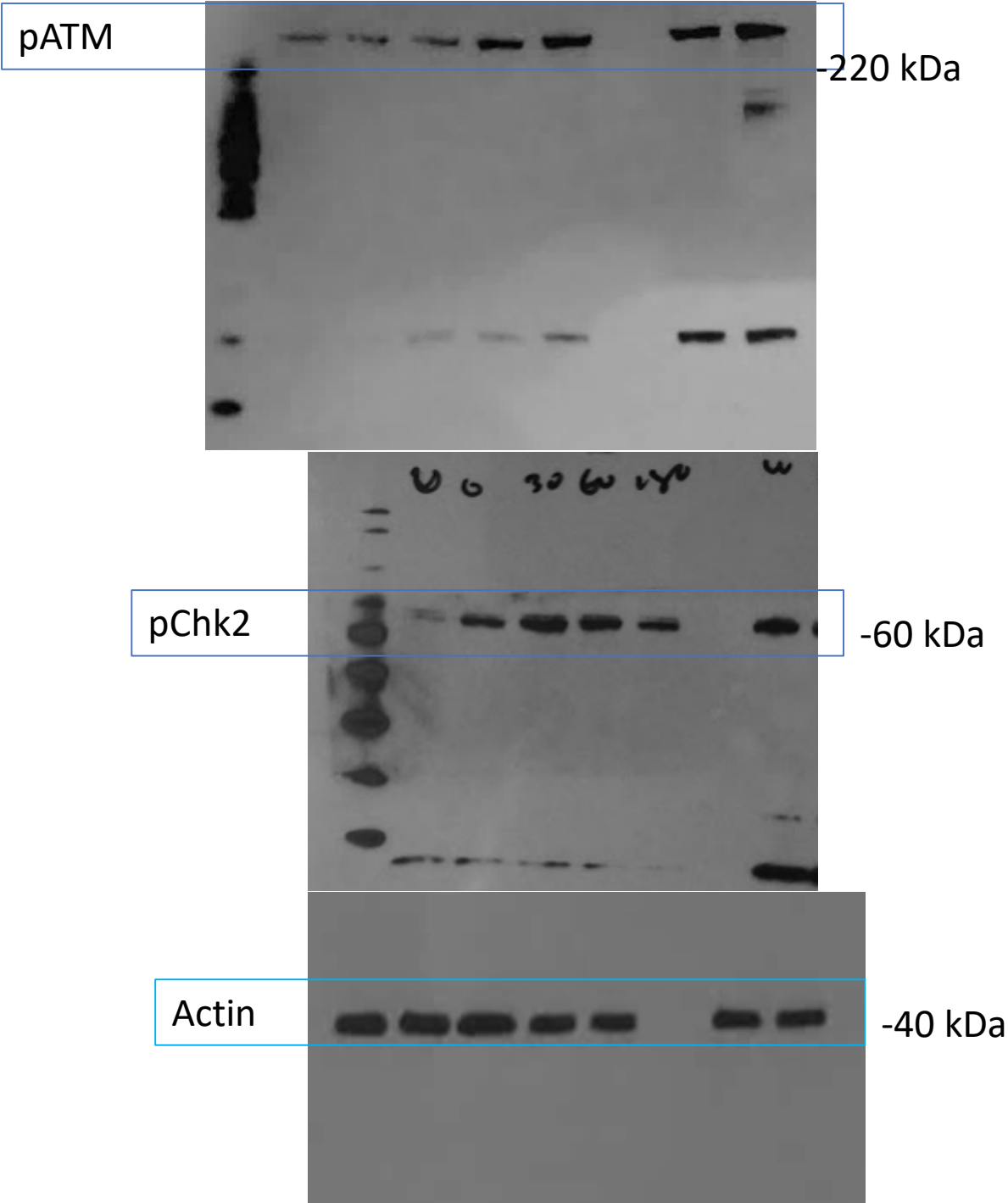

Fig 3e

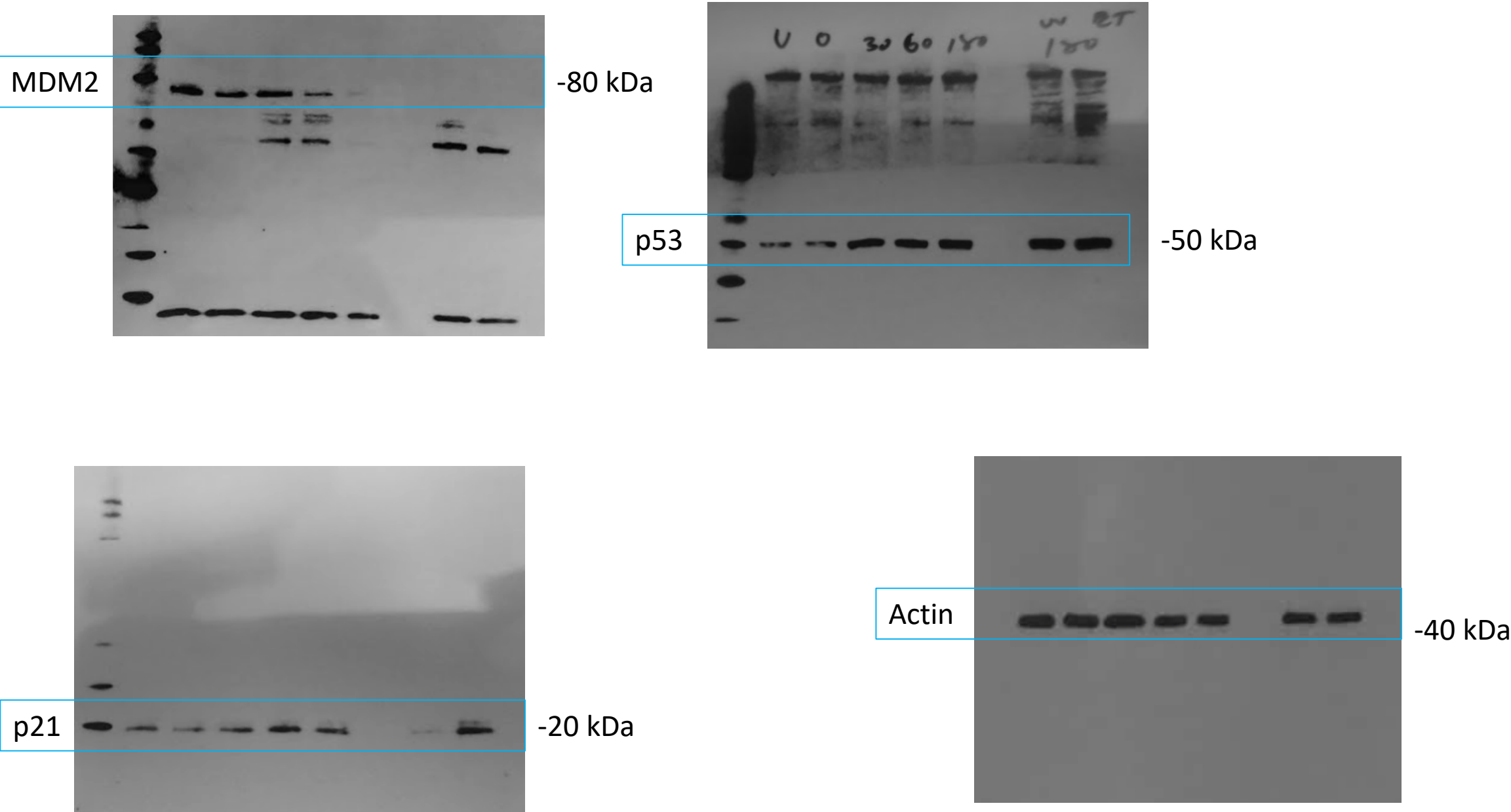

Fig 3g

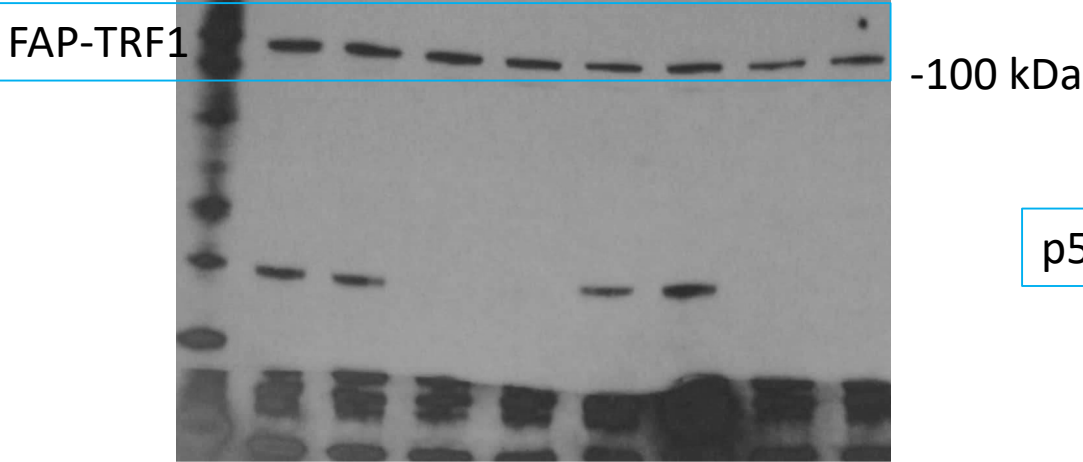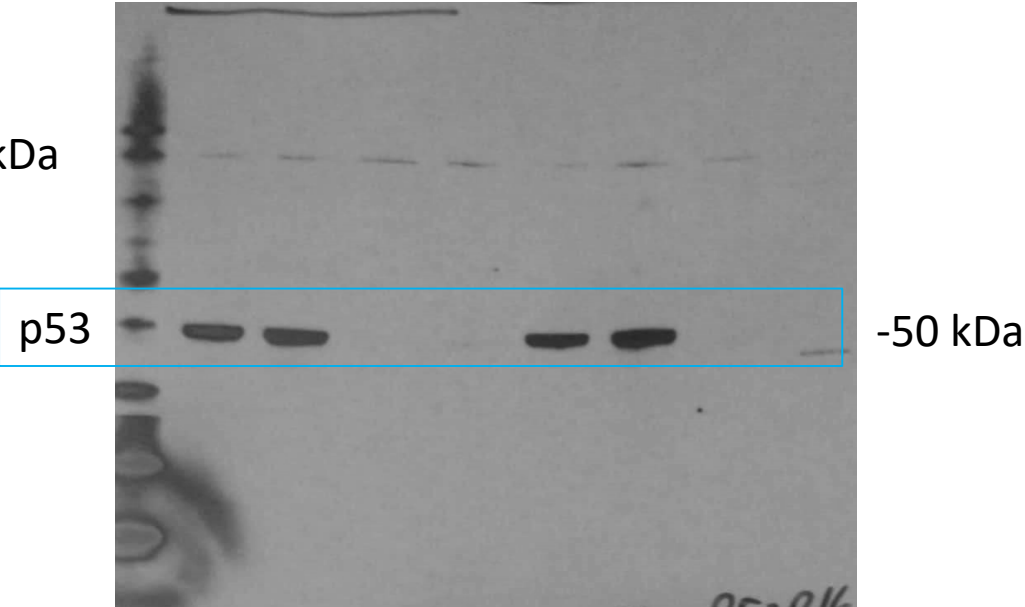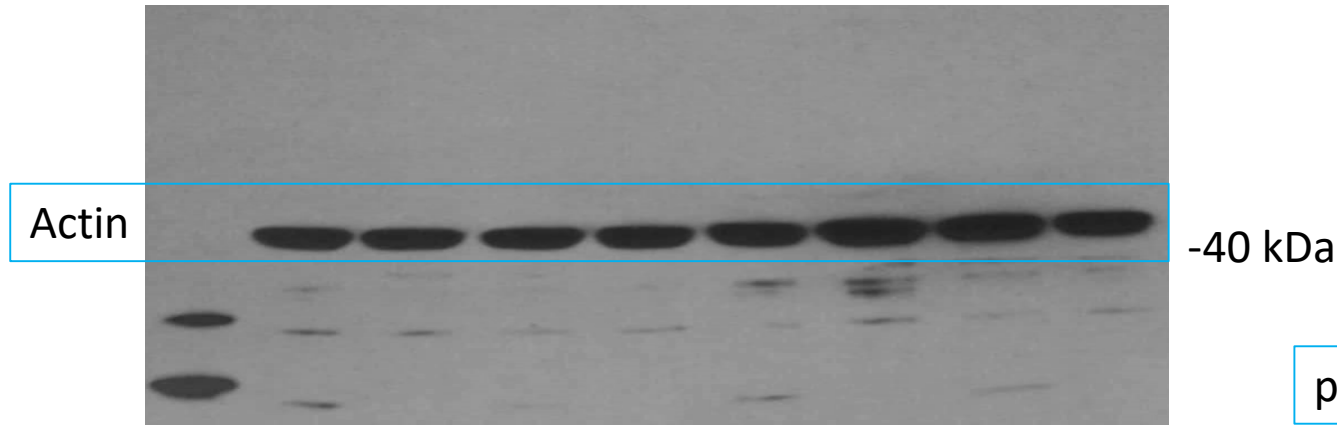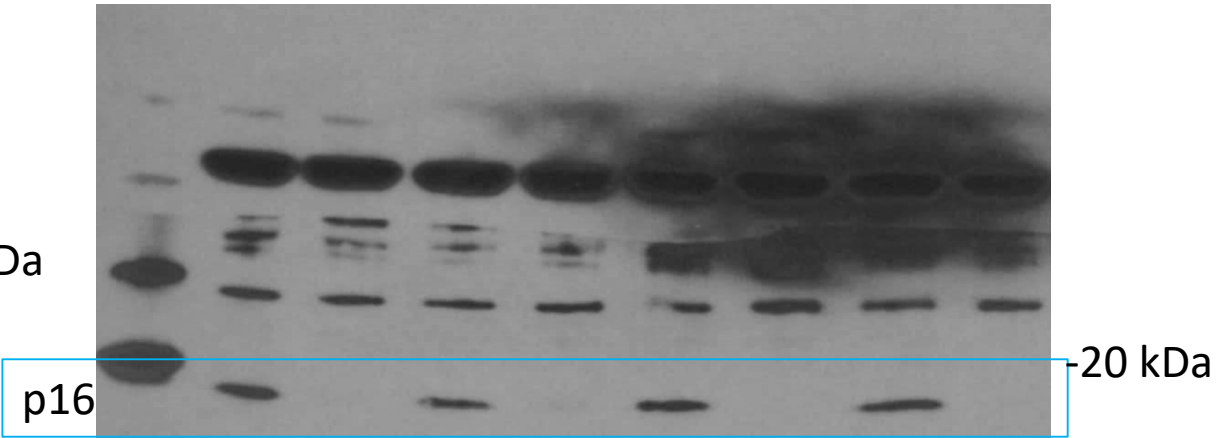

Supplement: Source Data Fig. 3 — Unprocessed western blots. [file 41594_2022_790_MOESM11_ESM.pdf]

FAP-TRF1 -100 kDa

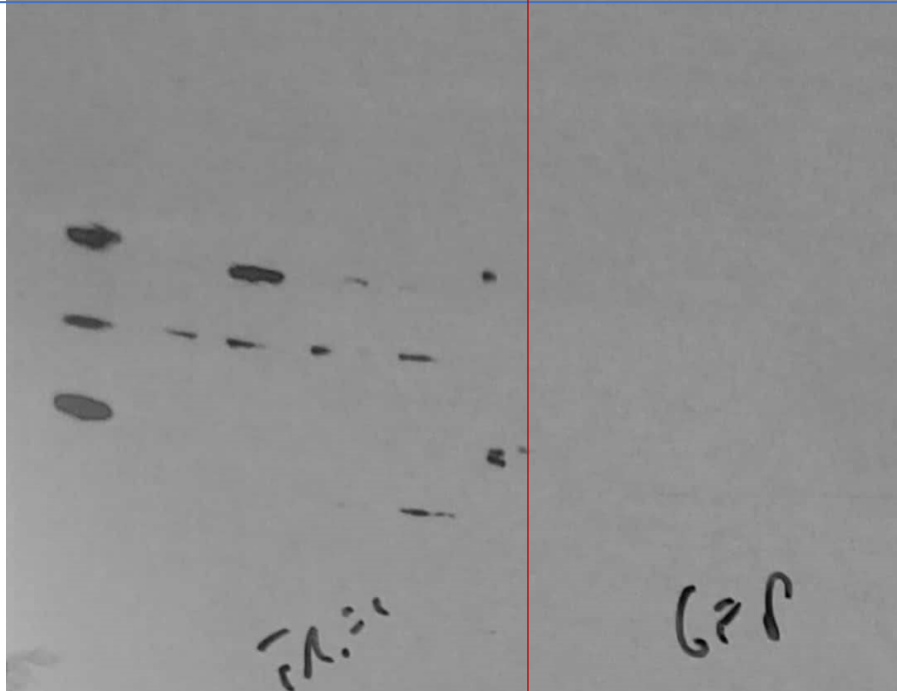

Anti-TRF1 (short)

Anti-GFP

TRF1  
(Long)

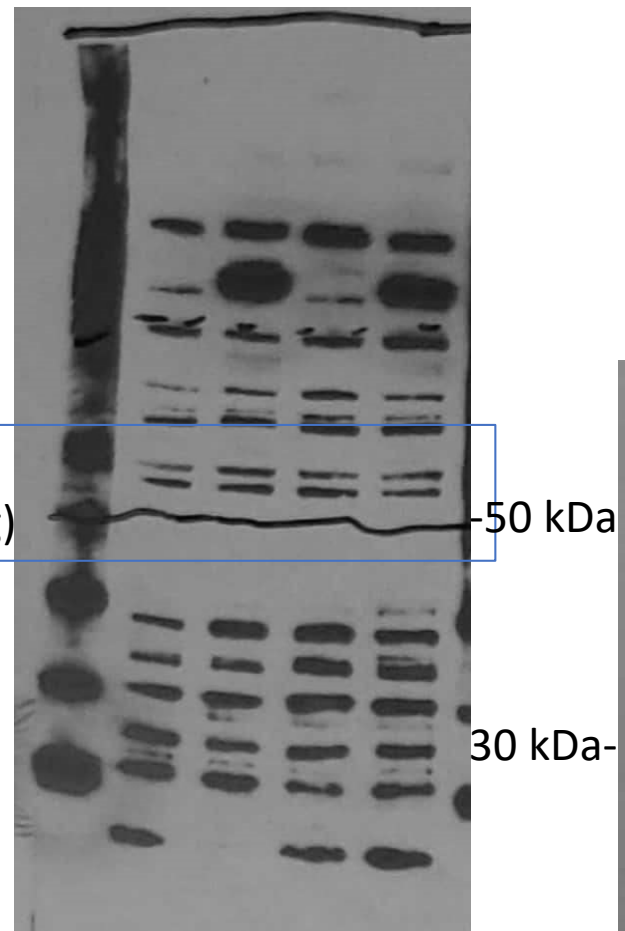

50 kDa

30 kDa

GAPDH

Supplement: Source Data Extended Data Fig. 1 — Unprocessed western blots. [file 41594_2022_790_MOESM16_ESM.pdf]

Extended Data Fig 5a

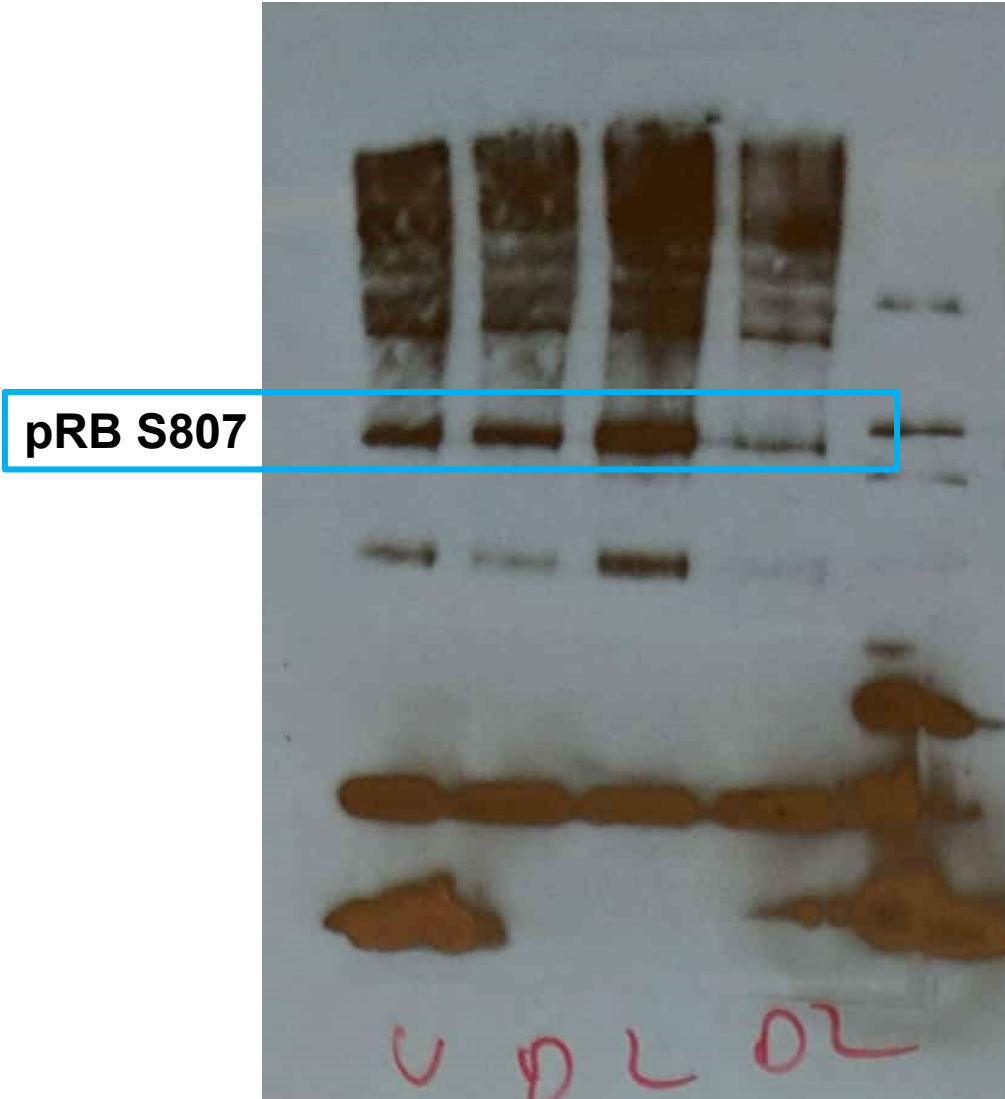

-120 kDa

Actin

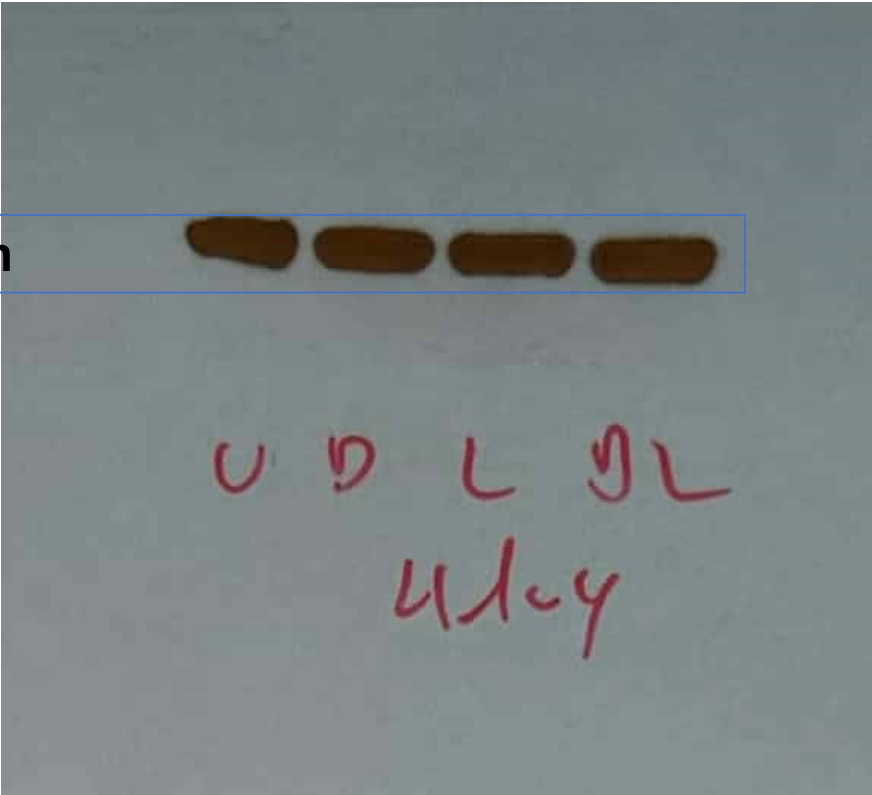

-40 kDa

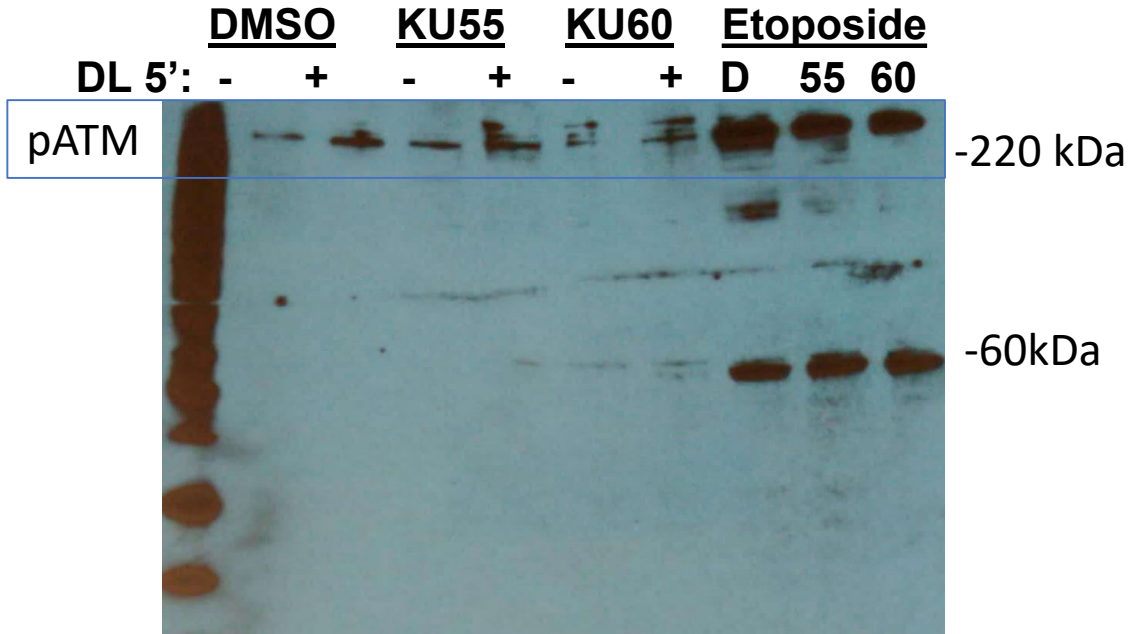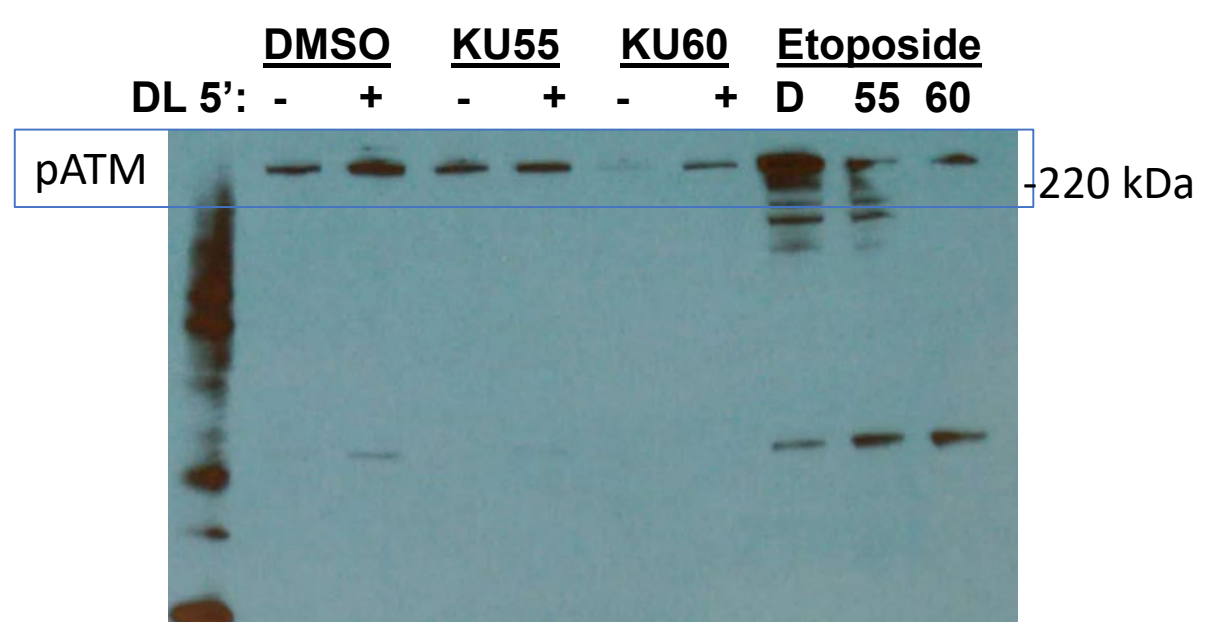

Extended Data  
Fig 5b (left)  
Fig 5c (right)

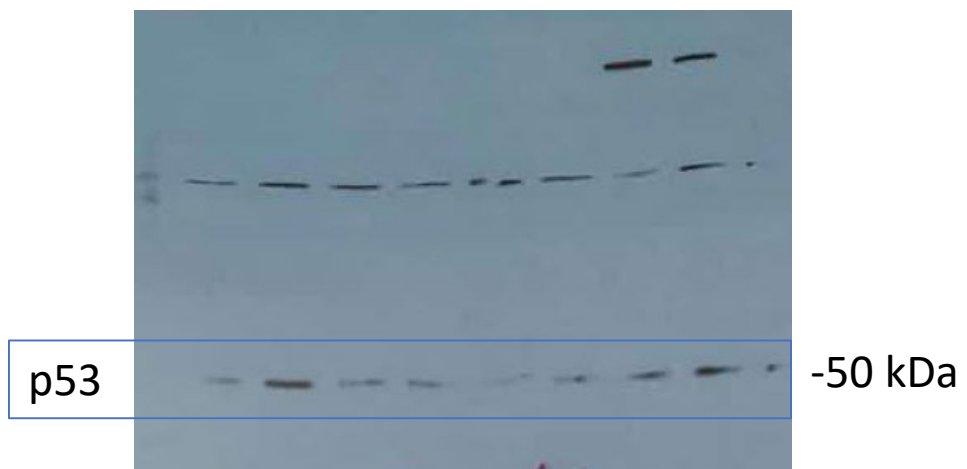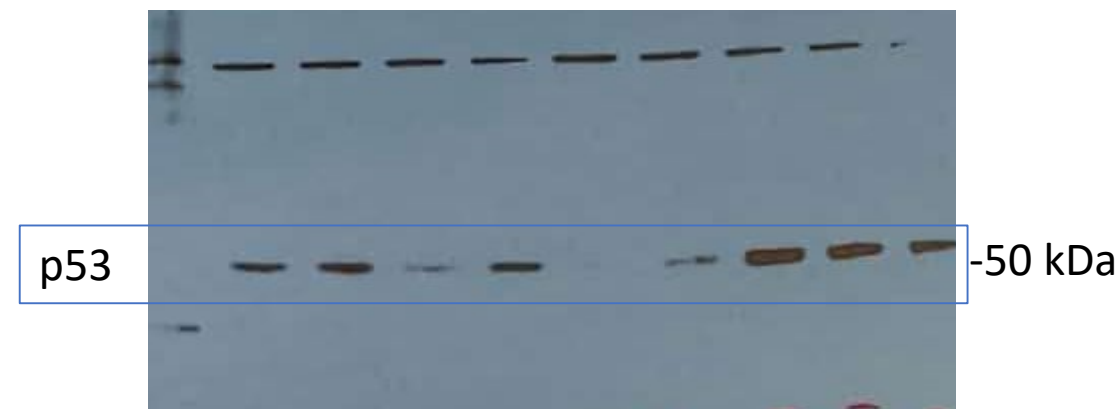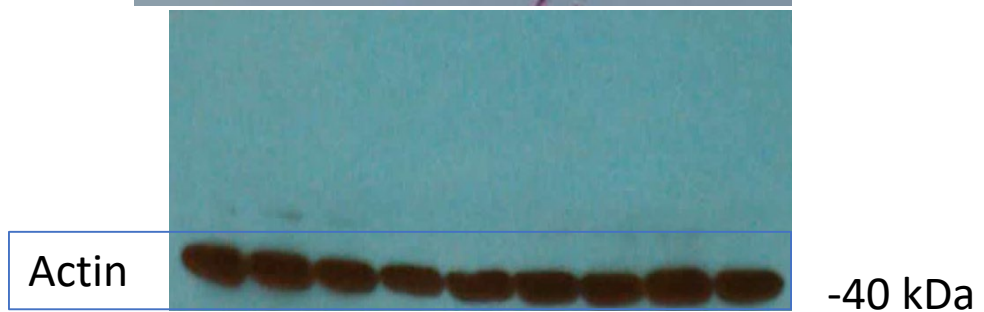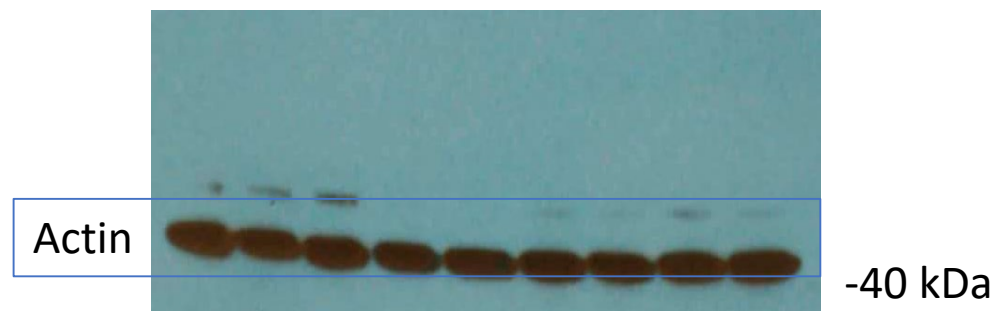

Supplement: Source Data Extended Data Fig. 5 — Unprocessed western blots. [file 41594_2022_790_MOESM21_ESM.pdf]

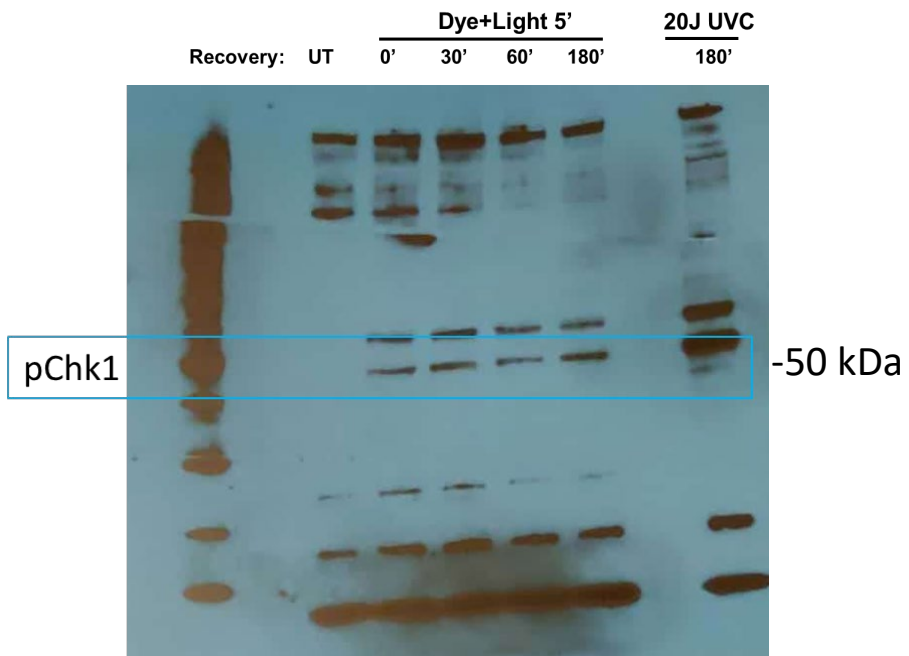

GAPDH

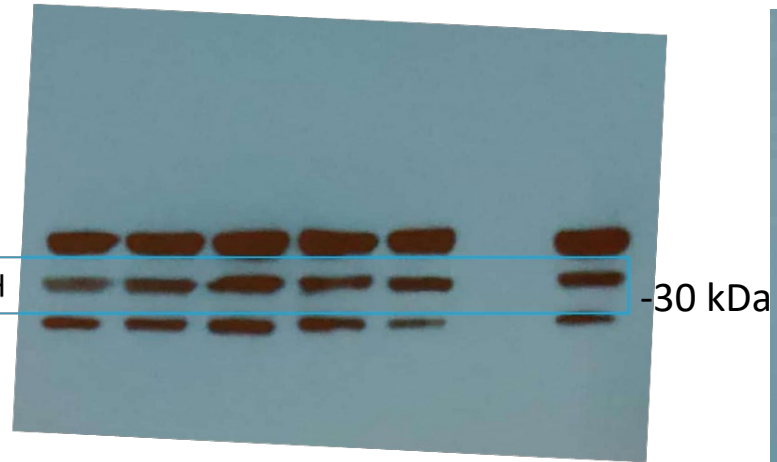

γH2AX

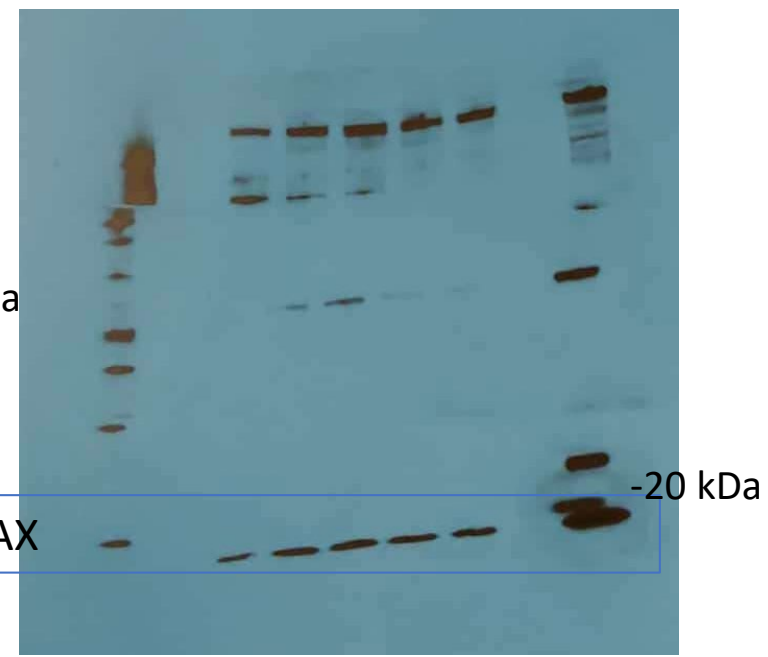

Total Chk1

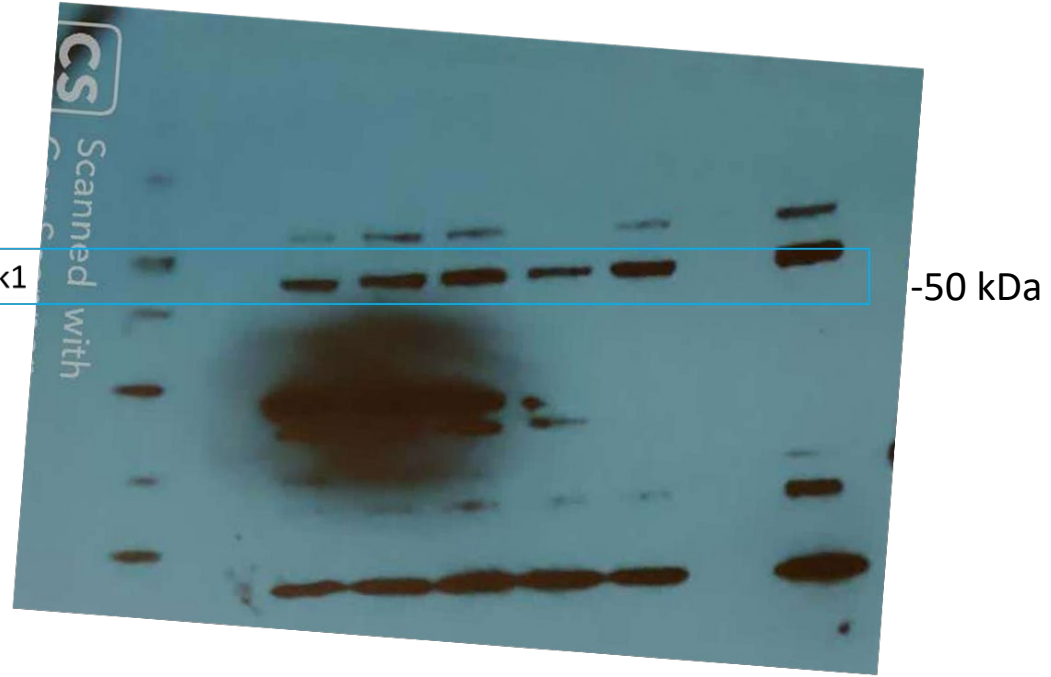

Total H2AX

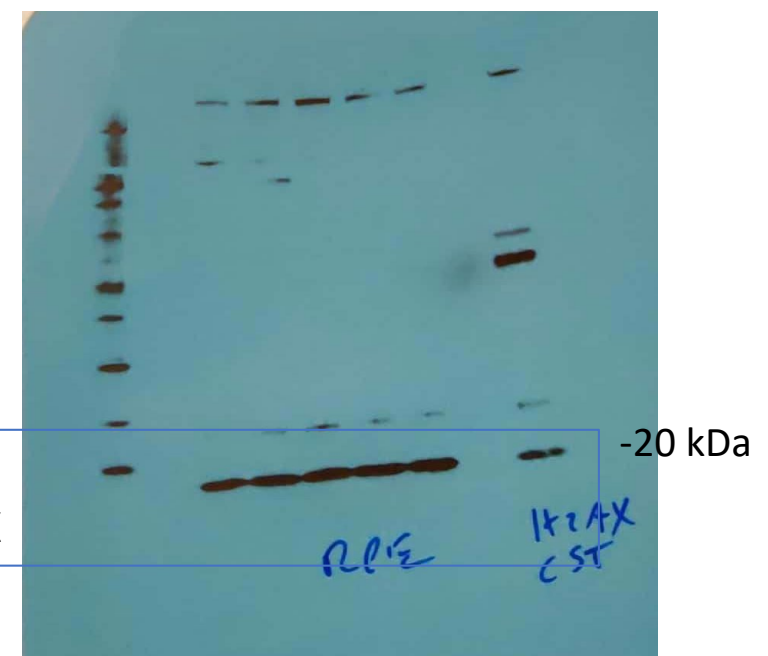

Supplement: Source Data Extended Data Fig. 9 — Unprocessed western blots. [file 41594_2022_790_MOESM26_ESM.pdf]

p53

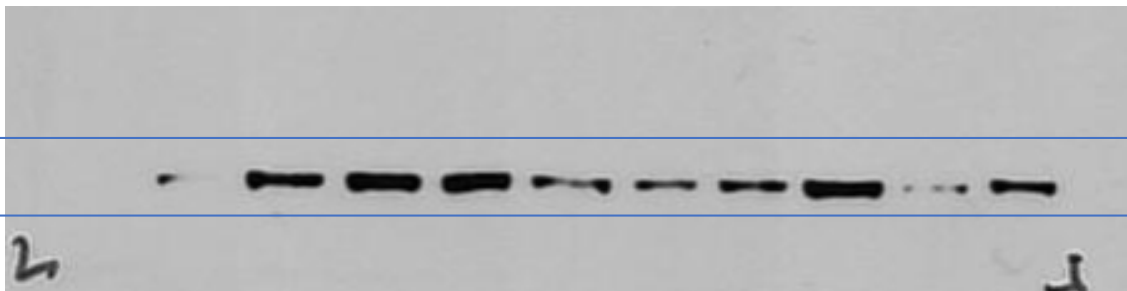

-50 kDa

pchk2

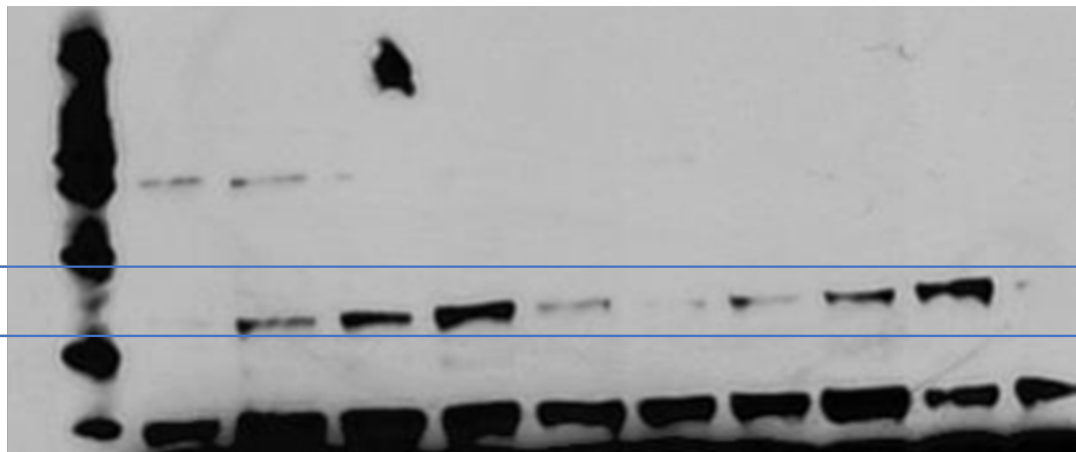

-60 kDa

Actin

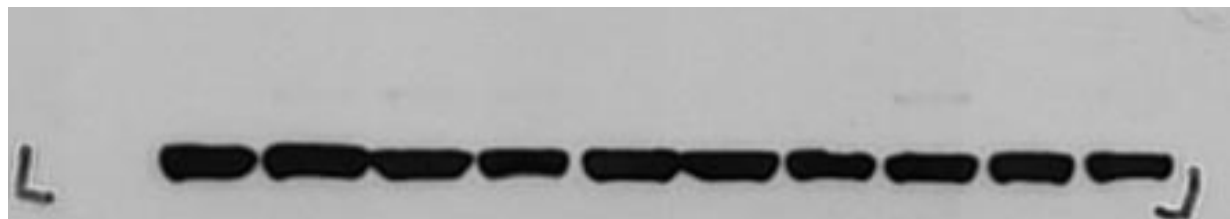

-40 kDa

Total chk2

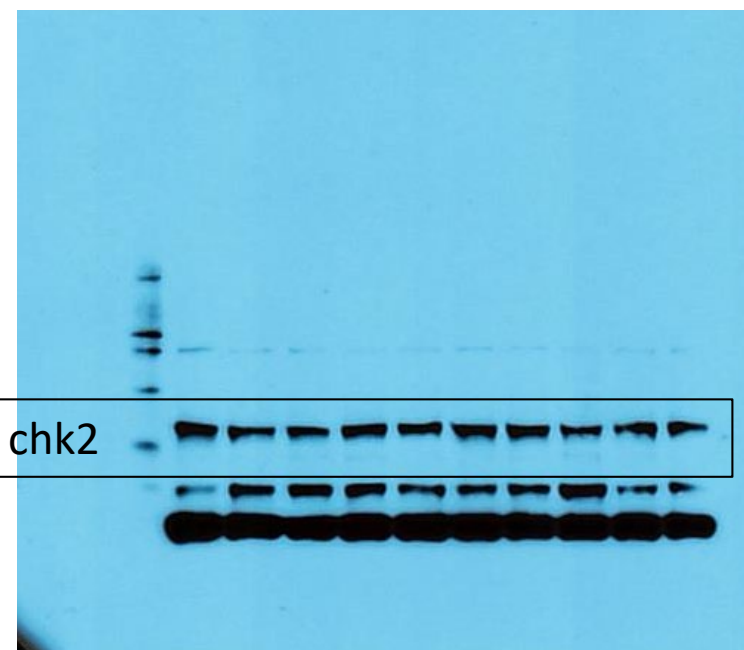

-60 kDa

Supplement: Source Data Extended Data Fig. 10 — Unprocessed western blots. [file 41594_2022_790_MOESM28_ESM.pdf]
